# Isothermal Crystallization Kinetics and Morphology of Double Crystalline PCL/PBS Blends Mixed with a Polycarbonate/MWCNTs Masterbatch

**DOI:** 10.3390/polym11040682

**Published:** 2019-04-15

**Authors:** Thandi P. Gumede, Adriaan S. Luyt, Agnieszka Tercjak, Alejandro J. Müller

**Affiliations:** 1 Department of Chemistry, University of the Free State (Qwaqwa Campus), Private Bag X13, Phuthaditjhaba 9866, South Africa; tpgumede66@gmail.com; 2 Central University of Technology, Department of Life Sciences, Private Bag X20539, Bloemfontein 9300, South Africa; 3 Center for Advanced Materials, Qatar University, P.O. Box 2713 Doha, Qatar; 4 Group ‘Materials + Technologies’ (GMT), Department of Chemical and Environmental Engineering, Faculty of Engineering, Gipuzkoa, University of the Basque Country UPV/EHU, Donostia-San Sebastián 20018, Spain; agatercjak@gmail.com; 5 Polymat and Polymer Science and Technology Department, Faculty of Chemistry, University of the Basque Country UPV/EHU, Paseo Manuel de Lardizabal 3, Donostia-San Sebastián 20018, Spain; 6 Ikerbasque, Basque Foundation for Science, Bilbao 48013, Spain

**Keywords:** PC/MWCNTs masterbatch, PCL/PBS blends, nanocomposites, morphology, conductivity, isothermal crystallization

## Abstract

In this work, the 70/30 and 30/70 w/w polycaprolactone (PCL)/polybutylene succinate (PBS) blends and their corresponding PCL/PBS/(polycarbonate (PC)/multiwalled carbon nanotubes (MWCNTs) masterbatch) nanocomposites were prepared in a twin-screw extruder. The nanocomposites contained 1.0 and 4.0 wt% MWCNTs. The blends showed a sea-island morphology typical of immiscible blends. For the nanocomposites, three phases were formed: (i) The matrix (either PCL- or PBS-rich phase depending on the composition), (ii) dispersed polymer droplets of small size (either PCL- or PBS-rich phase depending on the composition), and (iii) dispersed aggregates of tens of micron sizes identified as PC/MWCNTs masterbatch. Atomic force microscopy (AFM) results showed that although most MWCNTs were located in the PC dispersed phase, some of them migrated to the polymer matrix. This is due to the partial miscibility and intimate contact at the interfaces between blend components. Non-isothermal differential scanning calorimetry (DSC) scans for the PCL/PBS blends showed an increase in the crystallization temperature (*T*_c_) of the PCL-rich phase indicating a nucleation effect caused by the PBS-rich phase. For the nanocomposites, there was a decrease in *T*_c_ values. This was attributed to a competition between two effects: (1) The partial miscibility of the PC-rich and the PCL-rich and PBS-rich phases, and (2) the nucleation effect of the MWCNTs. The decrease in *T*_c_ values indicated that miscibility was the dominating effect. Isothermal crystallization results showed that the nanocomposites crystallized slower than the neat blends and the homopolymers. The introduction of the masterbatch generally increased the thermal conductivity of the blend nanocomposites and affected the mechanical properties.

## 1. Introduction

Global environmental issues and the realization that petroleum resources are finite have attracted considerable attention. Biodegradable polymers offer a potential solution to the environmental hazard posed by the accumulation of non-biodegradable plastics waste [1,2,3]. Different types of biodegradable polymers such as poly(ε-caprolactone) (PCL), poly(butylene succinate) (PBS), poly(lactic acid) (PLA), and poly(alkanoates) (PHA, PHB, PHBV) have been studied as potential biomaterials for a variety of applications. Amongst the mentioned biodegradable polymers, poly(butylene succinate) (PBS) has been favored in most applications because of its relatively good melt processability, biodegradability, and acceptable strength and modulus, which is closely comparable to those of the widely-used polyethylene (PE) and polypropylene (PP) [2,3]. Despite some advantages, the PBS biopolymer suffers from some disadvantages such as brittleness, which results in a low elongation at break.

It is well known that blending two or more polymers can result in a desirable combination of properties, which are often absent in the parent components. Blending offers advantages such as cost effectiveness and less time-consumption compared to the development of new monomers as a basis for new polymeric materials [4]. In order to improve the toughness of PBS, several authors blended it with PCL [5,6,7]. Generally, the authors reported that the tensile strength decreased as the PCL content increased in the blends, while the elongation at break and impact strength moderately increased with increasing PCL content. The decreased tensile strength indicated poor interfacial interaction between the blend phases. The higher elongation values showed improved ductility and toughness in the blends, due to the plasticization by PCL, which led to improved chain mobility and energy absorbed by the material before fracturing.

PBS/PCL blends are, however, immiscible as evidenced by the composition independent glass transition temperatures and the biphasic melt, which leads to poor interfacial adhesion and macrophase separation [7]. A number of studies investigated the effect of adding copolymers (poly(ethylene oxide)-block-poly(propylene oxide)-block-poly(ethylene oxide) (PEO-PPO-PEO) and poly(butylene succinate-*co*-ε-caprolactone) (P(BS-*co*-CL)) and thermoplastic soy meal (TSM) in order to improve miscibility, interfacial adhesion and the resultant mechanical properties of PCL/PBS blends [8,9,10]. In these studies, copolymers (PEO-PPO-PEO and P(BS-*co*-CL)) and thermoplastic soy meal (TSM) were used as compatibilizers. The compatibilizer was generally found to reduce particle sizes thereby increasing the surface contact area between the blend components. In some cases, the addition of the compatibilizer resulted in the disappearance of the spherical domains, exhibiting a rougher fracture surface than the blend without the compatibilizer. This apparently confirmed the effective reduction of interfacial tension and a significant improvement in compatibility and interfacial adhesion. Liu et al. [10] studied the effect of adding a P(BS-*co*-CL) compatibilizer in amounts up to 5 wt% in an 80/20 w/w PBS/PCL blend system. The modulus of elasticity, yield stress and fracture strain dramatically increased with the increasing compatibilizer content. This behavior was attributed to the improved interfacial compatibility in the presence of the compatibilizer. However, the properties declined significantly with further increases in the compatibilizer content. Can et al. [9] used PEO-PPO-PEO as a compatibilizer for PCL/PBS blends with different ratios. The E” value for PBS shifted to lower temperatures with the increasing PCL content, while the E” value for PCL slightly increased compared to that of neat PCL. This indicated an interaction and compatibility between the two polymers in the presence of the compatibilizer.

Recent reports [11,12,13] revealed that adding conductive carbon-based nano-fillers such as carbon nanotubes (CNTs) into PCL and PBS matrices can enhance some of the matrix properties to better levels than those of the copolymers or polymers filled with metal powders, as well as produce electrically conductive materials with better mechanical properties. This is due to their low density, inertness and better compatibility than metal powders with most polymers. CNTs have shown to have greater potential than any other carbon-based nano-fillers (i.e., carbon black (CB), carbon nanofibres (CNF), and graphite) because of their unique one-dimensional structure with good electrical conductivity, as well as excellent mechanical and thermal properties [14,15,16]. Furthermore, it was shown that the localization of carbon nanotubes in the PCL/PBS blends affected the material properties.

He et al. [11] synthesized double-crystalline PBS-*mb*-PCL multiblock copolymers and a series of PBS-*mb*-PCL/MWCNTs nanocomposites by solvent casting from solution. The dispersion of MWCNTs incorporated within a single polymer matrix (PBS/MWCNTs, PCL/MWCNTs) or within the copolymer (PBS-*mb*-PCL/MWCNT) was studied. The MWCNTs exhibited a much finer dispersion morphology in the PBS/MWCNTs nanocomposite than in the PCL/MWCNTs nanocomposite. The selective dispersion of MWCNTs in the PBS component improved the strength of the material without deteriorating its ductility. In the case of the PBS-*mb*-PCL immiscible multiblock copolymers, there was a refined microphase separation structure, while for the PBS-PCL/MWCNTs copolymer nanocomposites, the situation was more complex.

Our previous work on blending a PC/MWCNTs masterbatch with either the PCL or with PBS, evidence partial miscibility between PC and PCL in one case, and between PC and PBS in the other case. Therefore, these two blend nanocomposites exhibited two phases, a PC-rich phase and a PCL-rich phase in the case of the PCL/(PC/MWCNTs Masterbatch) and also two PC-rich and PBS-rich phases in the case of the PBS/(PC/MWCNTs Masterbatch). However, the extent of miscibility was different for each system. The polar component surface energy, interfacial tension and isothermal crystallization results indicated that the MWCNTs disperse better into the PBS-rich phase than in the PCL-rich phase.

In this paper, MWCNTs are dispersed into PCL/PBS blends through melt-mixing of the blend with a PC/MWCNTs masterbatch. The 70/30 and 30/70 w/w PCL/PBS blend ratios were selected because they exhibited the best mechanical properties of a range of investigated blend ratios. The structure and properties of the blend nanocomposites are correlated with the morphology of the PCL/PBS blends, and the dispersion of PC and MWCNTs in these blends. We also determine the influence of blending and nanofiller addition on the overall crystallization kinetics of the PCL and PBS components in the blends.

## 2. Experimental

### 2.1. Materials

A commercial polycaprolactone (PCL) (CAPA 6500, Johannesburg, South Africa) with a density of 1.1 g cm^−3^, a melting temperature of 58–60 °C, and a degree of crystallinity of ~35% was purchased from Southern Chemicals in South Africa. It has a weight-average molecular weight (*M_w_*) and number-average molecular weight (*M_n_*) of 113,400 g mol^−1^ and 73,620 g mol^−1^ respectively, and a polydispersity index of 1.54. It has a melt flow index (MFI) of 11.5 g/10 min (2.16 kg/190 °C).

A commercial poly(1,4-butylene succinate) (PBS), extended with 1,6-diisocyanatohexane, was purchased from Sigma-Aldrich (Johannesburg, South Africa). It has a density of 1.3 g cm^−3^ at 25 °C and a melting temperature of 120 °C. The weight-average molecular weight (*M_w_*) of PBS is 63,000 g mol^−1^ [17]. It has a melt flow index (MFI) of 20.9 g/10 min (2.16 kg/190 °C).

A conductive masterbatch based on 85% low viscosity polycarbonate (Makrolon^®^ 2205 grade, *M_w_* of 20,100 g mol^−1^ [18]) loaded with 15 wt% multiwalled carbon nanotubes (MWCNTs) (industrial grade NC7000), was obtained from Nanocyl (Sambreville, Belgium). It has a density of 1.175 g cm^−3^ and an MFI of 5.6 g/10 min, according to the specifications provided by the suppliers. The average diameter and length of the MWCNTs were respectively 10 nm and 3–4 µm. The MWCNTs contained more than 90% carbon and less than 10% metal oxide impurities.

The nanocomposites were prepared by melt-mixing in a twin-screw extruder (Thermo Scientific HAAKE Mini Lab II at the University of Pretoria, South Africa), which is operated under compressed air (100 rpm, 160 °C, 10 min). After extrusion, the samples were compression moulded at 160 °C for 5 min under 50 kPa using a hydraulic melt press. Table 1 shows the calculated weight percentages of the different components in each of the investigated nanocomposites.

### 2.2. Sample Characterization

Scanning electron microscopy (SEM) analyses were done in a TESCAN VEGA 3 scanning electron microscope at the Qwaqwa campus of the University of the Free State, Phuthaditjhaba, South Africa. The samples were sputter-coated with gold for 60 s to produce conductive coatings onto the samples. The acceleration voltage used was 15 kV.

Atomic force microscopy (AFM) experiments were performed on selected samples at room temperature using a Bruker Multimode 8 scanning probe microscope equipped with a Nanoscope V controller at the University of the Basque Country UPV/EHU, San Sebastian, Spain. The micrographs, with sizes in the range of 0.6–5 μm, were obtained in tapping mode by using microfabricated silicon tips/cantilevers (cantilever spring constant, *k* = 42 N m^−1^, and resonance frequency, *f*_0_ = 320 kHz, Bruker). Height and phase AFM images of lamellae and MWCNTs were collected simultaneously and subjected to a first-order plane-fitting procedure to compensate for the tilt. The height and phase AFM images were similar, and therefore only the phase AFM images will be reported in this paper. To obtain cross-sectional AFM images, the samples were cut using an ultramicrotome Leica Ultracut R with a diamond blade.

The contact angle measurements of the samples were conducted at room temperature on a surface energy evaluation system at the Qwaqwa campus of the University of the Free State, Phuthaditjhaba, South Africa, based on the sessile drop method. Five replicates for each sample were analyzed to ensure reproducibility of the results. Distilled water (H_2_O), and diiodomethane (CH_2_I_2_) were used as polar and non-polar solvents, respectively. The literature values of their surface energies are: H_2_O: γ^p^ = 50.7 mJ m^−2^ and γ^d^ = 22.1 mJ m^−2^; CH_2_I_2_: γ^p^ = 6.7 mJ m^−2^ and γ^d^ = 44.1 mJ m^−2^.

The melt flow index (MFI) of neat PCL and neat PBS were determined using a CEAST Melt Flow Junior at the Qwaqwa campus of the University of the Free State, Phuthaditjhaba, South Africa. Ten samples of each polymer were analyzed at 190 °C. The amount of sample, which flowed through the die over a period of 10 min under 2.16 kg weight, was determined in each case.

Dynamic mechanical analyses (DMA) were performed from −100 °C to the onset of melting of PBS, which is ~100 °C, in the bending (dual cantilever) mode at a heating rate of 3 °C min^−1^ and a frequency of 1 Hz.

Differential scanning calorimetry (DSC) analyses were performed using a heat flux Perkin Elmer DSC 6000 at the Qwaqwa campus of the University of the Free State, Phuthaditjhaba, South Africa under nitrogen flow (flow rate 20 mL min^−1^) to minimize degradation of the samples, and the instrument was calibrated at a heating rate of 10 °C min^−1^ using the onset temperatures of melting of indium and zinc standards, and the melting enthalpy of indium. The sample weight was almost exactly 5 mg in all cases.

For the non-isothermal DSC analyses, the samples were melted in the DSC for 3 min at 270 °C to erase any previous thermal history. The samples were then cooled at 20 °C min^−1^ from 270 to −60 °C, and then heated at the same rate from −60 to 270 °C.

The isothermal crystallization experiments were performed by following the procedure recommended by Lorenzo et al. [19], in which isothermal crystallization temperatures (*T_c_*) are chosen where no crystallization occurred during the cooling step from the melt (performed at 60 °C min^−1^). For the PCL-rich samples, the samples were heated to 90 °C and kept at this temperature for 3 min to erase the thermal history. The samples were then cooled at 60 °C min^−1^ to the set isothermal *T_c_*. The samples were then kept at the respective *T_c_* for a crystallization time (*t_c_*) until saturation was reached. Finally, the sample was heated from the *T_c_* to 90 °C at 20 °C min^−1^, to record the melting behavior of the isothermally crystallized sample. For the PBS-rich samples, the samples were heated to 270 °C and kept at this temperature for 3 min to erase the thermal history. The samples were then cooled at 60 °C min^−1^ to the set isothermal *T_c_*. The samples were then kept at the respective *T_c_* for a crystallization time (*t_c_*) until saturation was reached. Finally, the sample was heated from the *T_c_* to 270 °C at 20 °C min^−1^, to record the melting behavior of the isothermally crystallized sample.

To determine the equilibrium melting temperatures, $T_{m}^{o}$, of the samples, the sample was heated at 20 °C min^−1^ after isothermal crystallization in order to record the melting behavior of the isothermally crystallized polymer. The melting temperatures of the crystals, formed at different crystallization temperatures (*T_c_*), were recorded as the observed melting temperatures (*T_m(obs)_*). A Hoffman–Weeks extrapolation [20] was then applied by plotting *T_m(obs)_* against *T_c_* to observe the intersection of this line with another line with a slope equal to 1 (*T_m_* = *T_c_*).

The thermal conductivity measurements were performed using a Therm Test Inc. Hot Disk TPS 500 thermal constant analyser at the Qwaqwa campus of the University of the Free State, Phuthaditjhaba, South Africa. The instrument uses the transient plane source method. A 3.2 mm radius Kapton disk type sensor was selected for the analysis. The sample discs were 5 mm thick and 12 mm in diameter. The sensor was placed between two sample discs of the same composition. The measurements were done for 25 s in order to prevent the heat flow from reaching the boundary of the samples. Ten measurements were performed for each composition. The thermal conductivities are reported as average values with standard deviations.

The tensile testing analysis of the samples was carried out using an Instron 4301 universal testing machine at a cross-head speed of 10 mm min^−1^ at the Qwaqwa campus of the University of the Free State, Phuthaditjhaba, South Africa. The dumbbell shaped samples had a thickness of 1 mm, a gauge length of 20 mm and a width of 5 mm. The samples were tested at a controlled ambient temperature of 23 °C and 50% relative humidity. Three samples of each composition were tested and average values with standard deviations are presented.

## 3. Results and Discussion

### 3.1. Phase Morphology of PCL/PBS Blends and PCL/PBS/(PC/MWCNTs) Blend Nanocomposites

**Figure 1:**
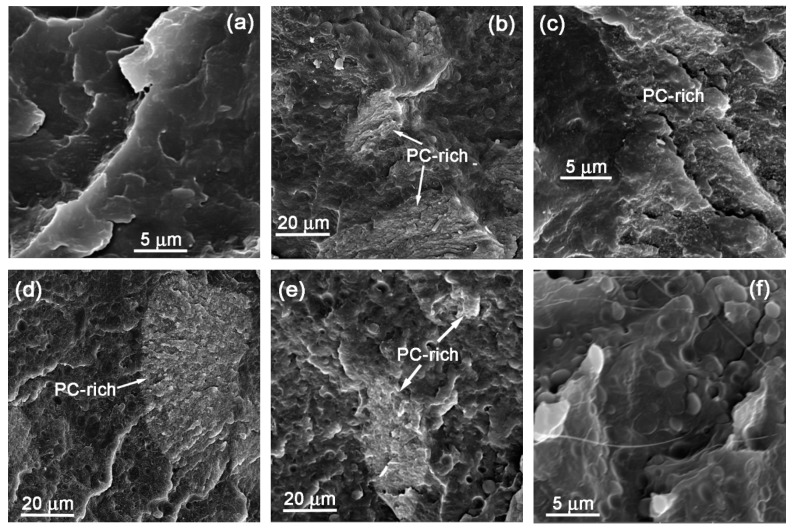
SEM images for (**a**) 30/70/0, (**b**) 28/65/(6/1), (**c**) 22/51/(23/4), (**d**) 51/22/(23/4), (**e**) 65/28/(6/1) and (**f**) 70/30/0 w/w PCL/PBS/(PC/MWCNTs) blend nanocomposites.

Phase morphology plays an important role in the mechanical behavior of polymer blends. The type of morphology and the sizes of dispersed phases in the polymer blends are important factors that determine the physical properties of these blends. In order to evaluate the morphology of the PCL/PBS blends and its filled nanocomposites, scanning electron microscopic (SEM) and atomic force microscopic (AFM) analyses were conducted. Figure 1 shows the SEM micrographs of the PCL/PBS blends and the PCL/PBS/(PC/MWCNTs) blend nanocomposites at different blend and masterbatch ratios.

The SEM images in Figure 1a,f show a sea-island morphology with discrete droplets of the minor phase in the matrix of the major phase, typical of immiscible polymer blends. The approximate diameters of the dispersed minor phases are shown in Table 2. It can be seen that the size of the minor phase for the 30/70 PCL/PBS blend (Figure 1a) is smaller than that of the 70/30 PCL/PBS blend (Figure 1f). There are several factors that determine the final particle size of the dispersed phases in polymer blends, such as surface energy, interfacial tension, polar character, blend composition, molar mass, viscosity ratio, and differences between the degree of crystallinity of the components in the blend, as well as time, shear stress, and temperature of mixing [21]. Amongst the mentioned factors, viscosity ratio plays a major role in the sizes of the dispersed phases [22].

**Figure 2:**
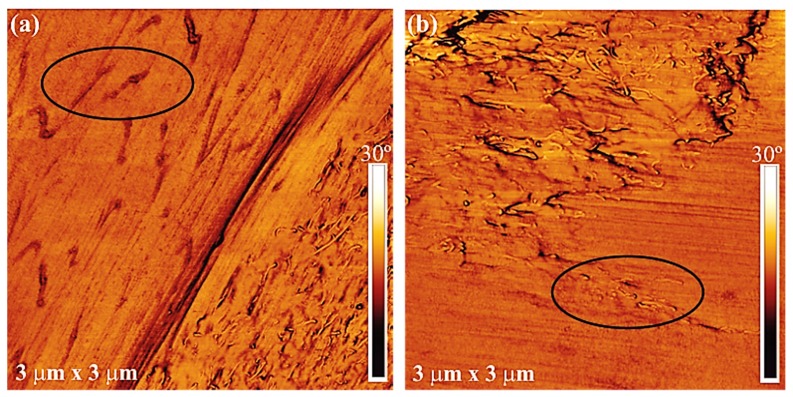
AFM images for the (**a**) 65/28/(6/1) and (**b**) 51/22/(23/4) w/w PCL/PBS/(PC/MWCNTs) blend nanocomposites.

In the case of the blends filled with the (PC/MWCNTs) masterbatch (Figure 1b–e), the SEM images show three phases: (i) The matrix (either PCL or PBS rich phase depending on the composition), (ii) dispersed polymer droplets of small size (either PCL or PBS rich phase depending on the composition), typically 3 μm or less, and (iii) dispersed phases of tens of micron sizes containing large concentrations of MWCNTs, which clearly is the (PC/MWCNTs) masterbatch, and which are indicated in Figure 1 with arrows. However, although most MWCNTs are located in the PC dispersed phases (Figure 1d,e), some of the MWCNTs (indicated with a black circle in the AFM images in Figure 2) migrated from the dispersed PC phase to the polymer matrix outside the dispersed phase. This is due to the partial miscibility and intimate contact at the interfaces between the PC-rich and PCL-rich phases, or the PC-rich and PBS-rich phases, as previously reported [23,24,25,26].

It is worth noting that when the PCL/PBS blends were loaded with 1 wt% MWCNTs, the sizes of the dispersed polymer droplets for the 65/28/(6/1) w/w PCL/PBS/(PC/MWCNTs) nanocomposite were smaller than those of the 28/65/(6/1) w/w PCL/PBS/(PC/MWCNTs) nanocomposite. When the PCL/PBS blends were loaded with 4 wt% MWCNTs, the sizes of the dispersed polymer droplets were smaller in the 22/51/(23/4) w/w PCL/PBS/(PC/MWCNTs) nanocomposite than those in the 51/22/(23/4) w/w PCL/PBS/(PC/MWCNTs) blend nanocomposite (Table 2). The smaller droplets indicate that there is compatibilization between the component phases. However, amongst the nanocomposites investigated in this paper, the 22/51/(23/4) PCL/PBS/(PC/MWCNTs) nanocomposite is the one giving the smallest particle sizes of the dispersed phase. This is due to the lower interfacial tension values between PBS and the PC/MWCNTs masterbatch compared to those between PCL and the PC/MWCNTs masterbatch (see Table 3). At equilibrium the particles will likely disperse in the phase where the affinity between the polymer and the nanoparticles is high.

The contact angles, total surface energies, as well as their dispersive and polar surface components, were calculated using the Owens-Wendt method [12,27,28] (Equations (1) and (2)).

(1) $$\gamma_{s}=\gamma_{s}^{d}+\gamma_{s}^{p}$$

(2) $$\gamma_{1}\left(1+cos\theta \right)=2\sqrt{\gamma_{s}^{d}.\gamma_{l}^{d}+\gamma_{s}^{p}.\gamma_{l}^{p}}$$

where *θ* is the contact angle, *γ* is the surface energy, the subscripts ‘*s*’ and ‘*l*’ respectively indicate solid and liquid, while ‘*d*’ and ‘*p*’ respectively indicate the dispersive and polar components. If the contact angle of at least two liquids, usually polar and nonpolar liquids with known $\gamma_{l}^{d}$ and $\gamma_{l}^{p}$ values, are measured on a solid surface, the $\gamma_{s}^{d}$ and $\gamma_{s}^{p}$ and the total surface energy ($\gamma_{s}$) of the solid can be calculated by using Equations (2) and (3) [29]. The interfacial tensions between the components in a blend were calculated from the surface energy measurement results using the geometric mean equation (Equation (3)) [12], and the wetting coefficient (Equation (4)) from the interfacial tensions.

(3) $$\gamma_{12}=\gamma_{1}+\gamma_{2}-2\sqrt{\gamma_{1}^{d}.\gamma_{2}^{d}+\gamma_{1}^{p}.\gamma_{2}^{p}}$$

where $\gamma_{12}$ = interfacial tension between components 1 and 2 in the blend, $\gamma_{1}$ and $\gamma_{2}$ are the total surface energies of components 1 and 2, $\gamma_{1}^{d}$ and $\gamma_{2}^{d}$ are the dispersive surface energies of components 1 and 2, and $\gamma_{1}^{p}$ and $\gamma_{2}^{p}$ are the polar surface energies of the components in the nanocomposites. The wetting coefficient, w_α_, is calculated by using Equation (4).

(4) $$w_{\alpha }=\frac{\gamma_{polymer\;B-Filler}-\gamma_{polymer\;A-Filler\;}}{\gamma_{polymerA-polymerB}}$$

where γ_polymerB-Filler_ is the interfacial tension between polymer B and the filler, γ_polymerA-Filler_ the interfacial tension between polymer A and the filler, and γ_polymerA-polymerB_ the interfacial tension between polymers A and B. The value of the wetting coefficient is normally used to determine where the filler is likely expected to disperse. If ω_α_ < −1, the particles are predicted to be localised in polymer B, if ω_α_ > 1, they are dispersed in polymer A, and if the value of ω_α_ is between −1 and 1, the nanoparticles are likely dispersed on the interface between the two polymers in the blend. In rare cases where the particles are dispersed in both the interface and one of the phases, the third condition does not apply, so that a negative ω_α_ indicates dispersion of the particles in polymer B as well as the interface, and a positive ω_α_ indicates dispersion of the particles in polymer A and on the interface [30,31]. The results are summarised in Table 3 and Table 4.

Table 3 shows that the polar component of the surface energy, γ^p^, for the masterbatch is closer to that of PBS. In terms of the interfacial tension values reported in Table 4, it can be seen that the interfacial tension between PCL and (PC/MWCNTs) (0.97 mN m^−1^) is larger than that between PBS and (PC/MWCNTs) (0.36 mN m^−1^). These results and the −0.26 mN m^−1^ wetting coefficient value suggest that the nanotubes would preferably disperse better in the PBS-rich phase.

Similar results were reported by He et al. [11], who introduced MWCNTs to double crystalline PBS/PCL blends using solution mixing and solvent casting processes. The MWCNTs exhibited a much finer dispersion morphology in the PBS/MWCNT nanocomposite than in the PCL/MWCNT nanocomposite. This was attributed to the wetting coefficient data for MWCNTs introduced into the binary copolymer, which was calculated as 1.73 (harmonic-mean equation) or −3.43 (geometric-mean equation). According to the authors, this meant that the MWCNTs were selectively distributed in the PBS phase.

Taking into account the results presented so far, we can conclude that a fair number of MWCNTs diffused from the PC-rich phase into the PCL-rich and PBS-rich phases, although one would expect, from the interfacial tension results, to find more MWCNTs in the PBS-rich phase. The majority of the MWCNTs were, however, still confined to the PC-rich droplets, despite the partial miscibility of the blends, and the intimate phase boundaries between the different components in the blends.

The ternary morphology obtained for the blends with a PC/MWCNT masterbatch, and especially the large sizes of the PC/MWCNTs phases, is obviously not the best as stress transfer may not be ideal, even when the phase boundaries obtained are small. Tuning of the morphology to obtain better mixing will have to be studied by varying extrusion conditions, screw configurations and processing variables in general. However, such a study was outside the scope of the present work.

### 3.2. Dynamic Mechanical Analysis (DMA)

Table 5 shows the glass transition temperatures obtained from the storage modulus (*E*’), loss modulus (*E*”) and tan δ curves. The DMA curves can be found in the Supplementary Information (see Figures S1 and S2). The addition of the masterbatch to the PBS-rich phase blend shows an increase in the glass transition temperature (*T_g_*). This is due to the partial miscibility between the PC-rich and PBS-rich phases. In the case of the PCL-rich phase, there is an increase in the *T_g_* values of the PCL in the nanocomposites, although there is no specific trend after the addition of the masterbatch. This suggests that the PBS-rich phase is more miscible with the PC than the PCL-rich phase.

### 3.3. Non-Isothermal DSC

**Figure 3:**
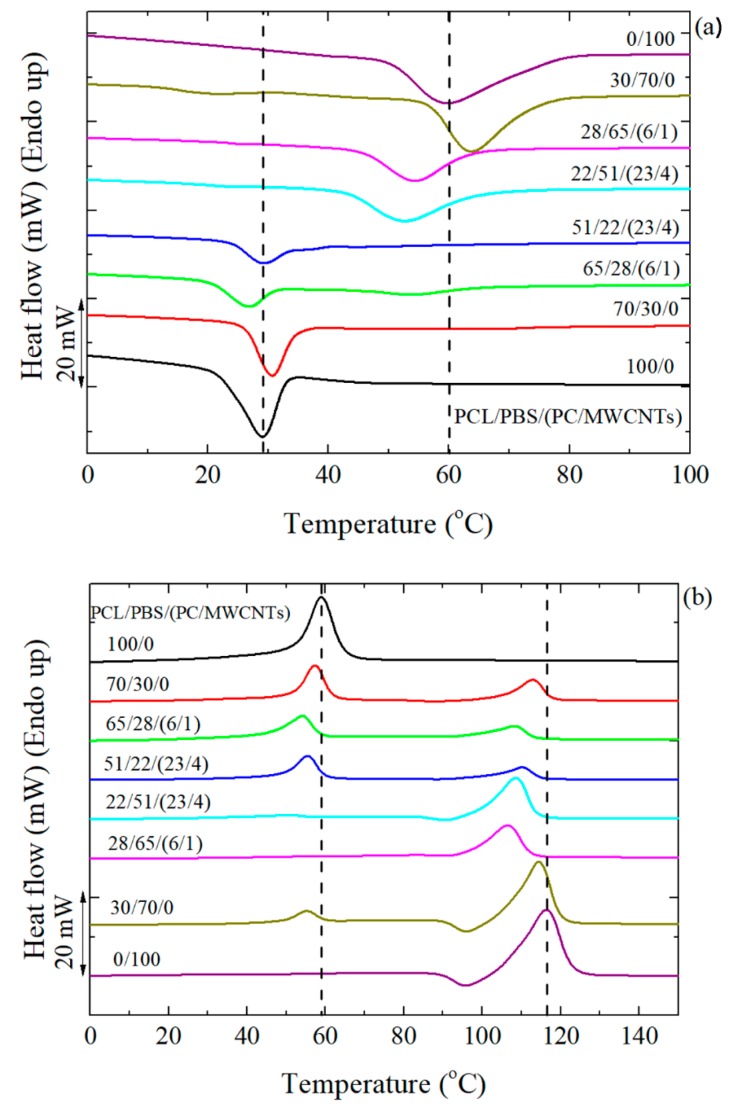
(**a**) Cooling and (**b**) second heating curves for the PCL/PBS blends and their nanocomposites.

Figure 3a shows the DSC cooling scans after erasing the thermal history, and Figure 3b the subsequent heating scans performed at 20 °C min^−1^ for the different investigated samples. Since PBS crystallizes first, its crystallization peak appears at about 60 °C, followed by the crystallization peak of the PCL component at 30 °C.

When 30 wt% PCL was added to 70 wt% PBS, there was a shift in the crystallization temperature (*T_c_*) of PBS to higher temperatures. This indicates a nucleation effect of the PBS component in the blend, which can only be produced by a transfer of impurities from the PCL to the PBS component, as the PCL is molten at the relevant temperatures for PBS crystallization [32]. In the case of adding 30 wt% PBS to 70 wt% PCL, there was no significant shift in the *T_c_* value of the PCL component. This is because the PCL/PBS blends are immiscible as evidenced by the composition independent glass transition temperature and the biphasic melt [7]. Other authors studied the miscibility behavior of a related poly(ε-caprolactone)/poly(propylene succinate) (PCL/PPSu) blend. The PCL/PPSu blends were found to show a very limited miscibility in the molten state, since the polymer-polymer interaction parameter (χ_12_) was −0.11 [33].

When the PCL/PBS blends were loaded with different amounts of PC/MWCNTs masterbatch, the *T_c_* of the PBS-rich nanocomposites shifted to lower temperatures than those of the neat PBS and the blend with PBS as the major phase. The same is true for the PCL-rich nanocomposites, although the change was less significant. This is due to the competition between two effects: (1) The partial miscibility of the PC-rich, the PCL-rich, and the PBS-rich phases, and (2) the nucleation effect of the MWCNTs. Miscibility between the different components in the blend tends to decrease the values of *T_c_* (as high *T_g_* PC chains are being solubilized in the PBS-rich phase and to a lower extent in the PCL-rich phase) while the nucleation effect tends to increase the *T_c_* values. In this case, since the MWCNTs and PC were simultaneously added, the miscibility effect dominates because of the decrease in the *T_c_* values of the nanocomposites.

In the subsequent heating scans shown in Figure 3b, the melting temperatures (*T_m_*) of the 70/30 and 30/70 PCL/PBS blends show very little variation compared to the homopolymers. Normally, when the blends are immiscible, the *T_m_* is not affected. In the case of the filled nanocomposites, the *T_m_* values decreased (more especially in the PBS-rich nanocomposites) compared to the neat materials and the blends. The decrease in the melting temperature values is due to miscibility effects between the PC-rich and the PCL-rich or PBS-rich phases. The PCL melting peak in the 28/65/(6/1) PCL/PBS/(PC/MWCNTs) nanocomposite is almost invisible. This is probably due to the PCL partial miscibility with the more rigid PC chains, making it difficult for PCL to crystallize, therefore forming fewer crystals.

**Figure 4:**
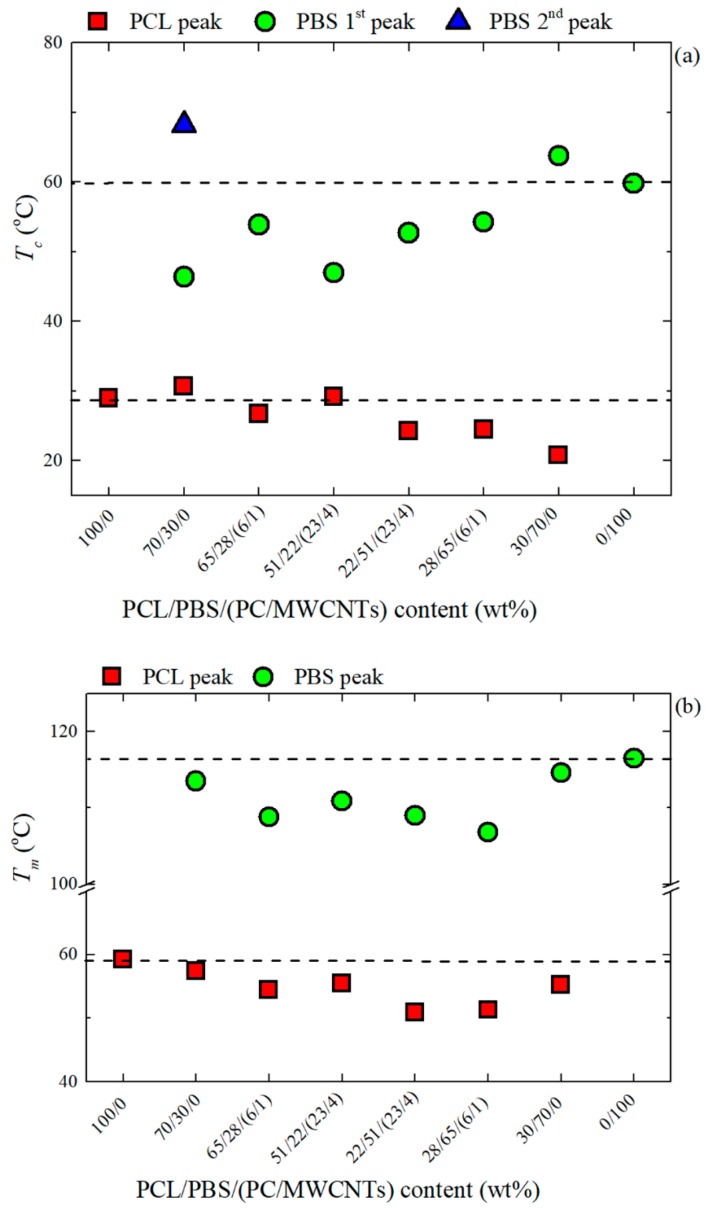
(**a**) Crystallization and (**b**) second heating melting temperatures for the PCL/PBS blends and the PCL/PBS/(PC/MWCNTs) nanocomposites.

To further examine the results presented in Figure 3, the *T_c_* and *T_m_* values were plotted as a function of sample composition in Figure 4. Figure 4a shows that the components in the 30/70 PCL/PBS blend are slightly more nucleated than the components in the 70/30 PCL/PBS blend. This is because of the smaller particle size of the PCL phase observed in the 30/70 blend (d_n_ = 0.4 microns) compared to that of the PBS component in the 70/30 blend (d_n_ = 1.3 microns), giving rise to a better dispersion and enhanced nucleation. The decrease in the *T_c_* values for the nanocomposites is attributed to the competition between partial miscibility with the PC-rich phase and the nucleation effect of the MWCNTs. It is clear that miscibility is the dominating factor.

In Figure 4b, the changes in *T_m_* are most significant when the masterbatch is added, because of the partial miscibility between the PCL-rich and PC-rich phases, as well as the PBS-rich and PC-rich phases. In the PBS-rich phase nanocomposites, the decrease in *T_m_* is more significant than in the PCL-rich nanocomposites. This is an indication that the PBS-rich phase is more compatible with the PC-rich phase than the PCL-rich phase, as indicated by the interfacial tension values in Table 4.

**Figure 5:**
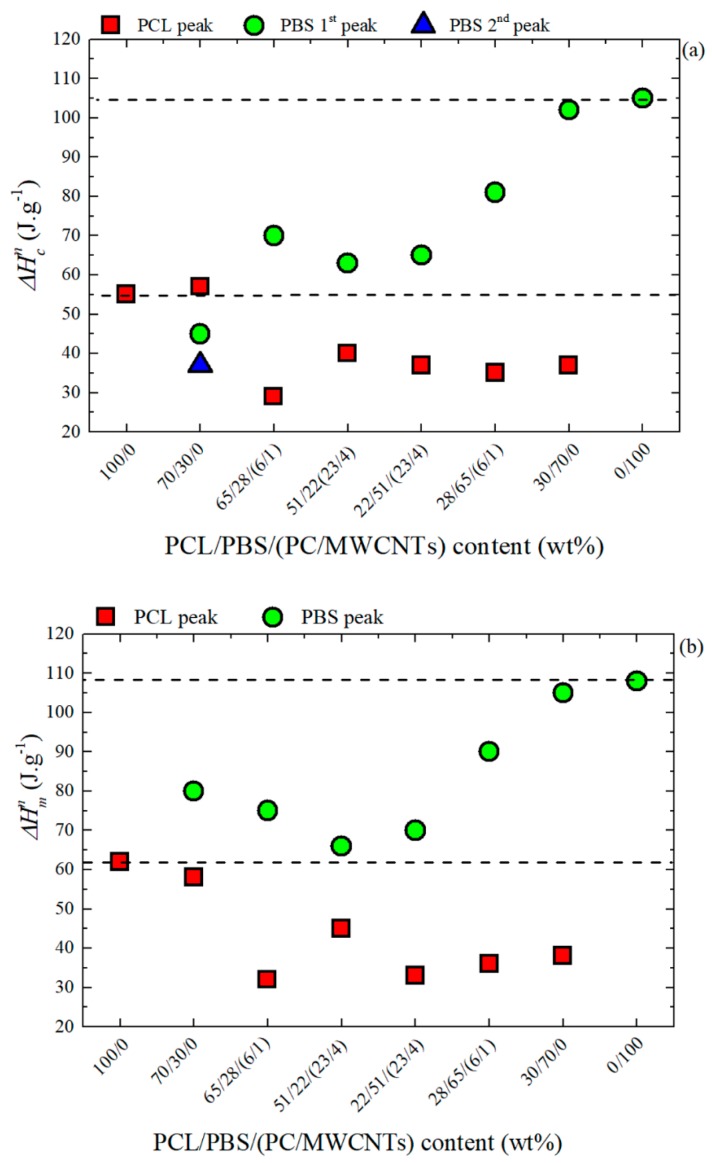
(**a**) Crystallization and (**b**) melting enthalpies for the PCL/PBS blends and the PCL/PBS/(PC/MWCNTs) nanocomposites.

Figure 5 shows the normalized crystallization and melting enthalpy values for all the samples. The normalized enthalpies for the PCL/PBS blends are almost the same as those of the homopolymers. This indicates that the total crystallinities of each of the two polymers are not significantly influenced by the presence of the other polymer in the blend. However, in the presence of the masterbatch, the normalized crystallization and melting enthalpy values for both polymers are lower than those of the neat polymers. This is due to the PC addition. When PC is added to the complex ternary blend, the resulting *T_g_* of the PCL-rich phase or the PBS-rich phase will be higher, as long as there is some partial solubility of PC chains in these respective phases. An effectively higher *T_g_* will make chain diffusion to the crystallization front more difficult. As previously discussed above, the DMTA results do show a significant increase of the *T_g_* for the PBS-rich phase when the PC/Masterbatch is added. In the case of the PCL-phase, the trend is not very regular with composition, but some slightly higher values are observed upon PC/Masterbatch addition depending on which criterion variable is used to determine the *T_g_* values.

The general trends observed in Figure 4 and Figure 5 indicate that the PBS-rich thermal properties are more affected by the addition of PCL and PC/Masterbatch than in the case of the PCL-rich phase (with the addition of PBS and PC/Masterbatch). This is consistent with the already mentioned notion that the miscibility between PBS and PC seems to be higher than that of PC and PCL, for the polymers employed in this work. It must be remembered that miscibility is a strong function of molecular weight.

### 3.4. Overall Isothermal Crystallization Studied by DSC

**Figure 6:**
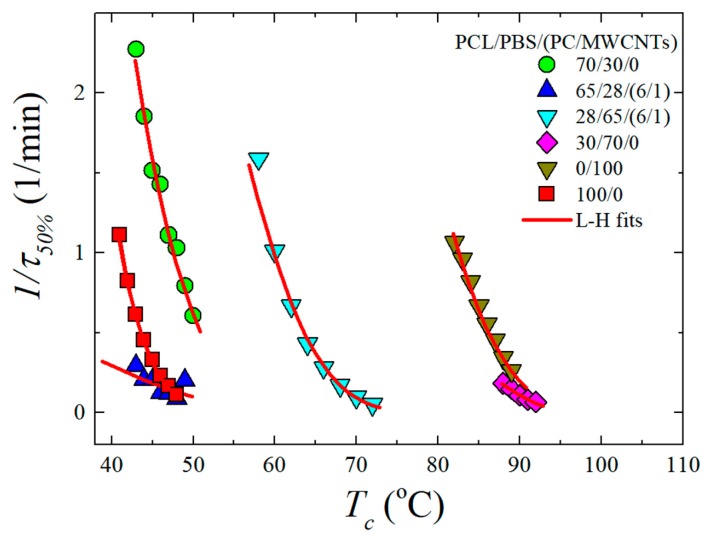
Half crystallization time (1/*t*_50%_) as a function of isothermal crystallization temperature (*T_c_*) for PCL (temperatures below 60 °C) and PBS (temperatures above 60 °C). The red solid lines represent fits to the LH theory.

The blend consists of two crystallisable components, i.e., PCL and PBS. Therefore, the influence of the PC/MWCNTs masterbatch on the isothermal crystallization kinetics of PCL (for temperatures below 60 °C) and PBS (for temperatures above 60 °C) is presented in this section. The inverse of the half crystallization time (1/*t*_50%_) as a function of the isothermal crystallization temperature (*T_c_*) for some of the investigated samples is shown in Figure 6. PBS clearly crystallizes at a higher temperature than PCL. The experimental data was fitted with the Lauritzen and Hoffman (LH) theory (see the Supplementary Information).

The PBS component in the neat 30/70 PCL/PBS blend shows a small change in the overall crystallization kinetics in comparison to the neat PBS. This is because the 30/70 PCL/PBS blend is immiscible as observed in the SEM images (Section 3.1). However, since the 30/70 blend shows nucleation effects, one would expect to see some increase in the overall crystallization rate, which includes both nucleation and growth components. But apparently, under isothermal conditions, the moderate nucleation effects observed in non-isothermal conditions (i.e., cooling from the melt), are not relevant.

When the PC/MWCNTs masterbatch is added to the 30/70 PCL/PBS blend, the crystallization rate of the PBS rich component in the blend is substantially lower than that of the neat PBS and the blend. This is because of the partial miscibility between the PC-rich and the PBS-rich phases. Miscible PC chains (that are more rigid than PBS chains) within the PBS-rich phases can decrease the rate of crystallization.

The PCL component in the 70/30 blend crystallizes faster than neat PCL. This is attributed to a nucleation effect of the previously crystallized PBS-rich phase. In order to perform the crystallization kinetics of the 70/30 blend, the sample is cooled from the melt and PBS can crystallize during such cooling. When the low *T_c_* temperatures needed to crystallize the PCL component are reached, the PBS has already finished crystallizing. Then the equipment is switched to the isothermal mode and the crystallization of the PCL component is measured. The blend with the masterbatch shows a slightly lower crystallization rate. This is due to the competition between the nucleation of the MWCNTs and the partial miscibility with PC. However, the miscibility effect obviously dominated, giving rise to a lower crystallization rate.

It is worth noting that the difference between the crystallization rate of neat PBS and the PBS-rich blend nanocomposite is much larger than that between the neat PCL and the PCL-rich blend nanocomposite. This suggests that the PBS-rich phase is more miscible with PC in comparison to the PCL-rich phase. A result consistent with the non-isothermal crystallization data presented above.

#### Fitting DSC Isothermal Data to the Avrami Model

**Figure 7:**
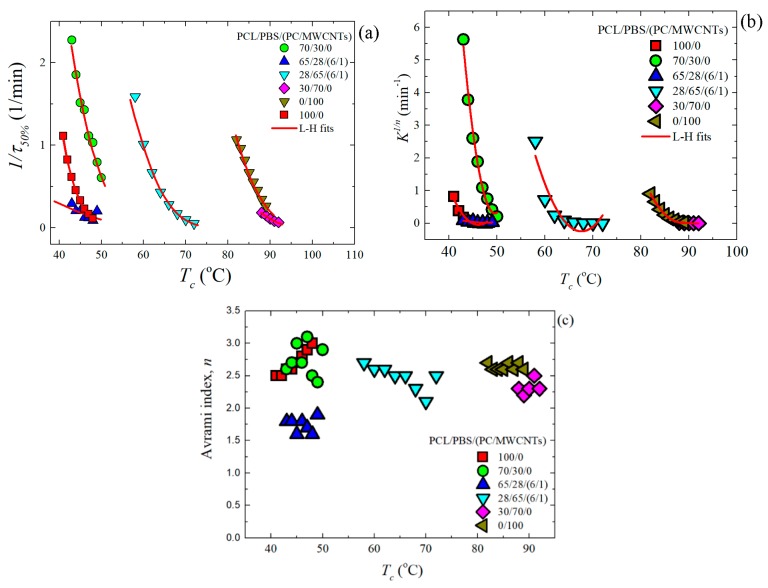
(**a**) Inverse of half crystallization times (1/*t*_50%_): (**b**) normalized crystallization constant of the Avrami model (*K*^1/*n*^), and (**c**) Avrami index (*n*) as a function of the isothermal crystallization temperature (*T_c_*) for all the samples. The red solid lines represent fits to the LH theory.

The data obtained during the isothermal crystallization experiments were analysed using the Avrami equation (Equation (5)) [34].

(5) $$1-V_{c}\left(t-t_{0}\right)=\exp(-K{\left(t-t_{0}\right)}^{n})$$

where *t* is the experimental time, *t*_0_ is the induction time, *V_c_* is the relative volumetric transformed fraction, *n* is the Avrami index, and *K* is the overall crystallization rate constant. The procedure used to perform the fittings to the data was developed by Lorenzo et al. [19]. The kinetic parameters for all the investigated samples are plotted in Figure 7 and tabulated in Table S1.

Figure 7a shows 1/*t*_50%_-values as a function of *T_c_*, the trend of which was explained earlier in the discussion (Section 3.3). A similar trend was obtained with the *K*^1/*n*^-values of the Avrami model (see Figure 7b), since this constant is also related to the overall crystallization rate. Figure 7c shows the Avrami index values, *n*, for all the samples. In the case of the PCL-rich samples, neat PCL and the 70/30 blend have *n*-values between 2.5 and 3.0. This is consistent with instantaneously nucleated spherulites. However, for its filled nanocomposite (65/28/(6/1) PCL/PBS/(PC/MWCNTs)), the value of *n* is between 1.5 and 2.0, which is approximately 2.0, indicating instantaneous axialites. It is known that the addition of a nucleating agent can cause the Avrami index values to change from 3.0 to 2.0 (as the dimensionality of growth can switch from 3D to 2D when the nucleation density is greatly enhanced) [19,34,35]. It was demonstrated in our previous study that the MWCNTs nucleate the PCL-rich phase [23]. The carbon nanotubes therefore affect the nucleation and the resultant morphology, while the crystallization rate is determined by the miscibility between the components.

The Avrami index values for neat PBS are within the range of 2.5–2.7, which is close to 3.0 indicating spherulitic morphology with instantaneous nucleation. For the blend and its nanocomposite, the *n*-values can be averaged to 2.3. This is because of a larger number of nucleation sites, which caused the formation of more crystals with an accompanying decrease in the probability of developing 3-D spherulites. 2D axialites instantaneously nucleated are probably formed in this case.

### 3.5. Thermal Conductivity

**Figure 8:**
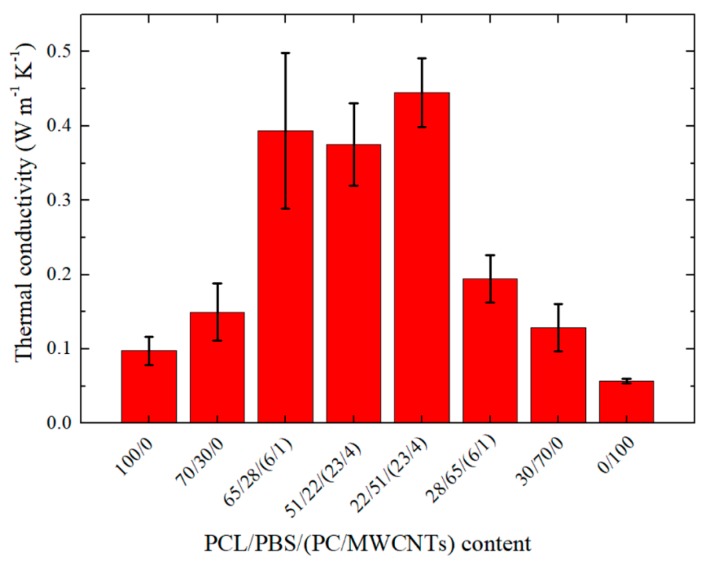
Influence of PC/MWCNTs masterbatch content on the thermal conductivities of the nanocomposites.

Figure 8 shows the thermal conductivity values for all the investigated samples. The homopolymers and the neat blends are typical insulators, hence their low thermal conductivity values (between 0.07 and 0.2 W m K^−1^).

The introduction of the masterbatch increased the thermal conductivity of the blend nanocomposites. This is because the carbon nanotubes have high thermal conductivities (in the range between 650 and 10,000 W m K^−1^) [36]. However, the thermal conductivity value of the 28/65/(6/1) sample is significantly lower than those of the other nanocomposites. The reason for this is probably the large number average particle diameter (*d*_n_ = 3.1 microns) of the dispersed phase that gives rise to a weaker interaction. This increased the acoustic impedance, which resulted in a large thermal contact resistance at the interface and a reduction in the thermal conductivity value of this nanocomposite.

In the case of the 4 wt% MWCNTs containing nanocomposites, the 22/51/(23/4) nanocomposite has a slightly higher thermal conductivity than the 51/22/(23/4) nanocomposite. The reason for this is probably the smaller and better dispersed MWCNTs particles in the nanocomposite. The smaller dispersed particles introduced more phonon scattering interfaces at the boundaries and facilitated better phonon transport due to the larger thermal contact areas.

### 3.6. Tensile Properties

**Figure 9:**
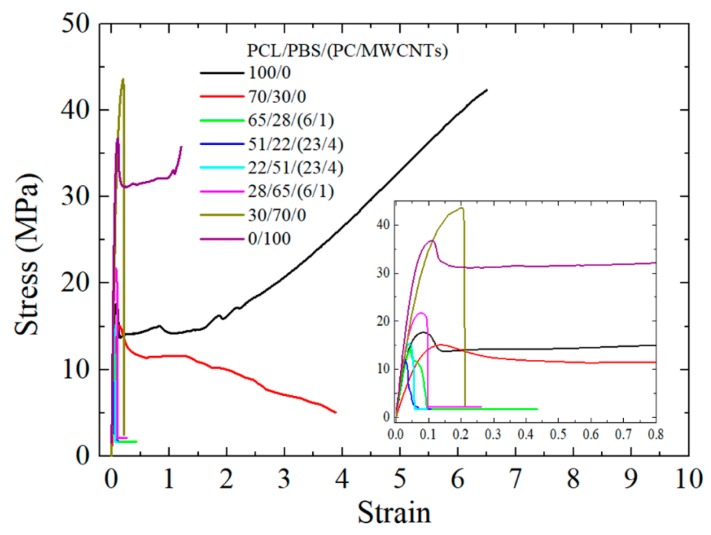
Stress-strain curves for neat PCL, neat PBS, PCL/PBS blends and its filled nanocomposites.

Figure 9 shows typical stress-strain curves obtained for all the samples, and Table 6 summarizes the tensile testing results. The Young’s modulus of the PCL/PBS blends is lower than that of the homopolymers for both PCL and PBS. This is associated with the weak interaction between the two polymers. The addition of the PC/MWCNTs masterbatch to the PCL/PBS blends generally showed little change in the Young’s modulus of the nanocomposites, taking into account the experimental error indicated by the standard deviation values. This may be due to the miscibility effect.

The stress and strain at break of the samples show a decrease after blending and after the incorporation of the masterbatch (Table 6). This is the result of the formation of the sea-island morphology, and in particular the very large PC-rich dispersed phase (with MWCNTs within it) acting as a stress concentration region. Cracks nucleate at the PC-rich phases and grow to produce earlier fracture. Yielding was only observed for the PCL-rich samples (due to the much higher ductility of PCL), and its values decrease with PBS addition and the incorporation of the masterbatch. Improvements in compounding techniques during extrusion must be explored in order to obtain better dispersions and smaller particles sizes. In this way, the mechanical properties may be improved.

## 4. Conclusions

Based on the SEM, AFM and surface property results, it can be concluded that the PCL/PBS blends are immiscible with discrete droplets of the minor phase within the matrix of the major phase. The nanocomposites prepared in this work are partially miscible. Three phases were formed: (i) The matrix (either PCL or PBS rich phase depending on the composition), (ii) dispersed polymer droplets of small size (either PCL or PBS rich phase depending on the composition), and (iii) the dispersed aggregates of tens of micron sizes which were clearly the PC/MWCNTs masterbatch. Due to the partial miscibility and the establishment of PC-rich, PCL-rich and PBS-rich phases, some of the MWCNTs migrated from the dispersed phase to the polymer matrix as evidenced by the AFM images.

Standard DSC measurements demonstrated an increase in *T_c_* for the PCL/PBS blends due to a nucleation effect. There are two possible explanations for this observation: (1) Transference of the impurities from the PCL phase to the PBS phase, and (2) since the PBS crystallizes first, the PCL droplets may have crystallized by surface induced nucleation on the interface with the PBS crystallized matrix and nucleated at the interphase. The nanocomposites showed a decrease in *T_c_* values. This was attributed to a competition between two effects: (1) The partial miscibility of the PC-rich with the PCL-rich and PBS-rich phases, and (2) the nucleation effect of the MWCNTs. The miscibility effect dominated because of the decrease in the *T_c_* values of the nanocomposites.

Isothermal crystallization experiments performed by DSC showed a decrease in the overall crystallization rates of the nanocomposites as a result of the competition between nucleating effect and miscibility. Since the PC-rich phase is partially miscible with the PCL-rich and PBS-rich phases, the miscible PC chains within PCL-rich and PBS-rich phases can decrease their rate of crystallization. The difference between the crystallization rate of neat PBS and the PBS-rich nanocomposite was much larger than that of the neat PCL and the PCL-rich nanocomposite. This confirmed that the PBS-rich phase is more miscible than the PCL-rich phase with the PC-rich phase.

The thermal conductivities of the nanocomposites were generally enhanced with the addition of the masterbatch, while the tensile properties of the nanocomposites did not improve (with respect to the neat blends), which was ascribed to the multiple phases present and the stress concentration effects provided by the large PC-rich droplets that included a high concentration of MWCNTs.

## Figures and Tables

**Table 1:** Percentages of the components in the nanocomposites.

PCL (%)	PBS (%)	PC (%)	MWCNTs (%)
100	0	0	0
70	30	0	0
65	28	6	1
51	22	23	4
22	51	23	4
28	65	6	1
30	70	0	0
0	100	0	0

**Table 2:** Particle sizes of the dispersed polymer phases in the PCL/PBS blends.

w/w PCL/PBS/(PC/MWCNTs)	Dispersed Polymer Droplets (d_n_ (µm)/d_v_ (µm)/D)
30/70/0	0.4/0.6/1.4
28/65/(6/1)	3.1/3.8/1.2
22/51/(23/4)	0.5/0.6/1.2
51/22/(23/4)	2.9/4.3/1.5
65/28/(6/1)	2.0/3.4/1.7
70/30/0	1.3/1.5/1.2

*d_n_*—number average diameter; *d_v_*—volume average diameter; *D*—particle size polydispersity; (PC/MWCNTs) is the masterbatch.

**Table 3:** Surface properties and melt flow index values for neat PCL, neat PBS and the (PC/MWCNTs) masterbatch.

Sample	Contact Angle/Degree		Surface Energy/mN m^−1^			MFI (g/10 min)
	H_2_O	CH_2_I_2_	Γ	γ^d^	γ^p^	
Neat PCL	59.7 ± 1.3	16.1 ± 0.2	58.4	48.8	9.6	11.5
Neat PBS	35.9 ± 0.7	14.8 ± 0.3	70.7	49.1	21.6	20.9
PC/MWCNTs masterbatch	47.6 ± 1.1	23.7 ± 1.0	63.2	46.6	16.6	5.6

γ = surface energy, γ^d^ = dispersive component of surface energy, γ^p^ = polar component of surface energy.

**Table 4:** Interfacial tensions and wetting coefficient of the investigated materials.

Component Pair	Interfacial Tension/mN m^−1^
PCL/PBS	2.38
PCL/(PC/MWCNTs)	0.97
PBS/(PC/MWCNTs)	0.36
Wetting coefficient (w_α_)	−0.26

**Table 5:** Transition temperatures (*T_g_*) obtained from *E’*, *E”* and tan δ curves for all the investigated samples.

PCL/PBS/(PC/MWCNTs)	*T_g_* (°C) from E’ vs. T		*T_g_* (°C) from E” vs. T		*T_g_* (°C) from tan δ vs. T	
**100/0**	−60.8	-	−47.9	-	−40.0	-
**70/30/0**	−58.2	−16.9	−49.0	−25.1	−43.4	−16.9
**65/28/(6/1)**	−54.4	−16.3	−41.2	−22.2	−36.6	−16.3
**51/22/(23/4)**	−55.8	−25.5	−44.9	−23.6	−43.9	−25.5
**22/51/(23/4)**	−53.8	−25.5	−48.6	−22.6	−49.2	−25.5
**28/65/(6/1)**	−56.3	−31.9	−50.2	−22.9	−49.6	−31.9
**30/70/0**	−52.4	−29.9	−51.2	−23.3	−49.0	−29.9
**0/100**	-	−35.5	-	−27.6	-	−35.5

**Table 6:** Summary of tensile testing results for all the samples.

w/w PCL/PBS/(PC/MWCNTs)	$\sigma_{y}/\mathrm{MPa}$	$\varepsilon_{y}/\%$	$\sigma_{b}/\mathrm{MPa}$	$\varepsilon_{b}/\%$	E/MPa
100/0	16 ± 2	14 ± 8	34 ± 13	578 ± 151	388 ± 29
70/30/0	15 ± 2	12 ± 13	12 ± 4	520 ± 142	274 ± 2
65/28/(6/1)	16 ± 1	4.4 ± 0.2	8 ± 1	7.7 ± 0.3	457 ± 39
51/22/(23/4)	-	-	11 ± 2	4.0 ± 0.8	432 ± 43
22/51/(23/4)	-	-	9 ± 3	5.8 ± 0.4	484 ± 35
28/65/(6/1)	-	-	19.7 ± 0.3	9 ± 1	448 ± 22
30/70/0	-	-	28 ± 8	50 ± 1	438 ± 11
0/100	-	-	38 ± 2	210 ± 110	579 ± 139

*σ_y_*—stress at yield; *ε_y_*—strain at yield; *σ_b_*—stress at break; *ε_b_*—strain at break; *E*—Young’s modulus.

## Supplementary Materials

The following are available online at https://www.mdpi.com/2073-4360/11/4/682/s1, Figure S1: DMA (a) storage modulus (E’), (b) loss modulus (E”), and (c) tan δ curves for neat PCL, neat PBS, PCL/PBS blends and the different nanocomposites, Figure S2: Glass transition temperatures (*T_g_*) obtained from storage modulus, loss modulus and tan δ curves, description of Lauritzen-Hoffman model, Table S1: Parameters from the isothermal crystallization kinetics analyses for the PCL/PBS blends and the PCL/PBS/(PC/MWCNTs) nanocomposites.
