# Supplementary material for: Isothermal Crystallization Kinetics and Morphology of Double Crystalline PCL/PBS Blends Mixed with a Polycarbonate/MWCNTs Masterbatch
Source: Polymers (Basel). 2019 Apr 15;11(4):682. doi: 10.3390/polym11040682 (PMC6523105; doi:10.3390/polym11040682)
Supplement: Supplementary file 1 [file polymers-11-00682-s001.pdf]

Supporting Information

Isothermal crystallization kinetics and morphology of double crystalline PCL/PBS blends mixed with a polycarbonate/MWCNTs masterbatch

Thandi P. Gumede[1,2], Adriaan S. Luyt[3]*, Agnieszka Tercjak[4] and Alejandro J. Müller[5,6]*

Dynamic mechanical analysis (DMA)

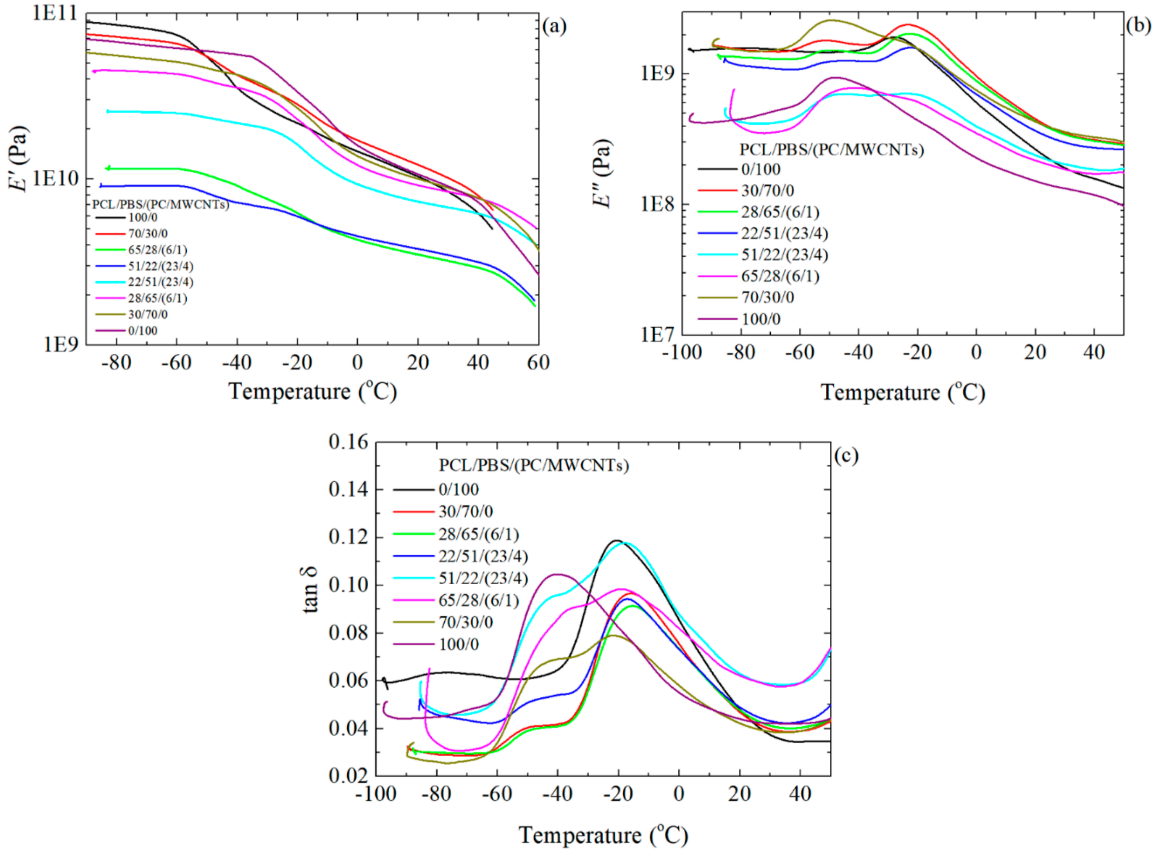

Figure S1 **DMA (a) storage modulus (E'), (b) loss modulus (E"), and (c) tan δ curves for neat PCL, neat PBS, PCL/PBS blends and the different nanocomposites**

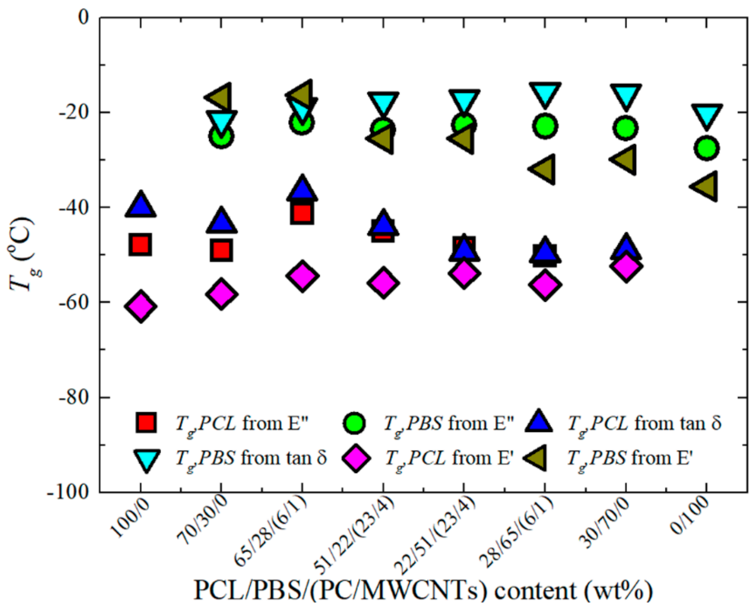

Figure S2 Glass transition temperatures (T_g) obtained from storage modulus, loss modulus and tan δ curves

Overall isothermal crystallization data analysed by the Lauritzen–Hoffman model

The overall crystallization kinetics is determined by contributions of nucleation and growth. The Lauritzen–Hoffman (LH) nucleation and growth theory can be applied to the isothermal crystallization kinetics data collected from DSC. Figure 6 shows solid lines that represent the mathematical fit of the LH theory, which can be applied to the DSC overall crystallization data according to Equation 1.

$$\frac{1}{\tau_{50\%}}(T) = \frac{1}{\tau_0}\exp\left(\frac{-U^*}{R(T_c - T_\infty)}\right)\exp\left(\frac{-K_g^\tau}{T_c \Delta T_f}\right), \qquad (1)$$

where $1/\tau_{50\%}$ is the inverse of the experimental half-crystallization time, $1/\tau_0$ is a pre-exponential factor that includes nucleation and growth, U^* is the activation energy for the transport of the chains to the growth front (a value of 1500 cal mol^{-1} is usually employed), R is the gas constant, T_c is the isothermal crystallization temperature (K), T_∞ is the temperature at which chain mobility ceases (usually taken as T_g – 30 K), ΔT is the supercooling ($T_m^o - T_c$), T_m^o is the equilibrium melting temperature calculated for each blend (see Table 6), and K_g^τ is a constant related to the energy barrier for crystallization and growth. The value of K_g^τ is given by Equation 2 according to the LH theory.

$$K_g^\tau = \frac{j b_0 \sigma \sigma_e T_m^0}{k \Delta h_f}, \quad (2)$$

where b_0 is the width of the chain, σ is the lateral surface free energy, σ_e is the fold surface free energy, k is the Boltzmann constant and Δh_f is the heat of fusion of a perfect crystal. The parameter j is determined by the operating regime and was taken as 2 for regime II (note that j is equal to 4 for regime I and III). The product $\sigma\sigma_e$ is obtained from the values of K_g^τ, according to Equation 7. Equations 3 and 4 allow the calculation of σ (and therefore σ_e) and q, the work done by the chain to form a fold.

$$\sigma = 0.1\Delta h_f \sqrt{a_0 b_0} \quad (3)$$

$$q = 2a_0 b_0 \sigma_e \quad (4)$$

where $a_0 b_0$ is the cross-sectional area of the chain. To obtain the parameters of the LH theory, the following values were used: T_g = 213 K, T_g – 30 K, ΔH = 163 J g^{-1}, a_0 = 4.52 Å, b_0 = 4.12 Å, p_c = 1.1 g cm^{-3}, U^* = 1500 cal mol^{-1}. The LH parameters for all the samples are tabulated in Table S1. Even though the LH theory is capable of fitting the collected data, the values of the parameters obtained do not follow logical trends. This could be due to the complexity of the multiphasic systems explored.

Table S1 Parameters from the isothermal crystallization kinetics analyses for the PCL/PBS blends and the PCL/PBS/(PC/MWCNTs) nanocomposites.

w/w PCL/PBS/(PC/MWCNTs)	T^o_m (K)	K^t_g x 10^5	σ	σ_e	q x 10^{-12}	R^2
100/0	373.0	4.52	8.08	474.90	1.75	0.9999
70/30/0	371.4	2.26	8.08	239.10	0.88	0.9914
65/28/(6/1)	369.8	1.57	8.08	166.31	0.61	0.9041
28/65/(6/1)	375.3	1.46	8.08	152.47	0.56	0.9960
30/70/0	379.8	0.33	8.08	33.47	0.12	0.9884
0/100	394.0	1.24	8.08	119.31	0.44	0.9972
